# Peroxisomes Are Highly Abundant and Heterogeneous in Human Parotid Glands

**DOI:** 10.3390/ijms24054783

**Published:** 2023-03-01

**Authors:** Christoph Watermann, Malin Tordis Meyer, Steffen Wagner, Claus Wittekindt, Jens Peter Klussmann, Sueleyman Erguen, Eveline Baumgart-Vogt, Srikanth Karnati

**Affiliations:** 1 Institute for Anatomy and Cell Biology II, Medical Cell Biology, Medical Faculty, Justus Liebig University Giessen, 35392 Giessen, Germany; 2 Department of Otorhinolaryngology, Head and Neck Surgery, Medical Faculty, Justus Liebig University Giessen, 35392 Giessen, Germany; 3 Department of Otorhinolaryngology, Clinic Center, 44137 Dortmund, Germany; 4 Department of Otorhinolaryngology, Head and Neck Surgery, Medical Faculty, University of Cologne, 50937 Cologne, Germany; 5 Institute for Anatomy and Cell Biology, Julius Maximilians University, 97070 Würzburg, Germany

**Keywords:** peroxisomes, parotid gland, human, catalase, differential expression, PSP, mRNA, immunofluorescence

## Abstract

The parotid gland is one of the major salivary glands producing a serous secretion, and it plays an essential role in the digestive and immune systems. Knowledge of peroxisomes in the human parotid gland is minimal; furthermore, the peroxisomal compartment and its enzyme composition in the different cell types of the human parotid gland have never been subjected to a detailed investigation. Therefore, we performed a comprehensive analysis of peroxisomes in the human parotid gland’s striated duct and acinar cells. We combined biochemical techniques with various light and electron microscopy techniques to determine the localization of parotid secretory proteins and different peroxisomal marker proteins in parotid gland tissue. Moreover, we analyzed the mRNA of numerous gene encoding proteins localized in peroxisomes using real-time quantitative PCR. The results confirm the presence of peroxisomes in all striated duct and acinar cells of the human parotid gland. Immunofluorescence analyses for various peroxisomal proteins showed a higher abundance and more intense staining in striated duct cells compared to acinar cells. Moreover, human parotid glands comprise high quantities of catalase and other antioxidative enzymes in discrete subcellular regions, suggesting their role in protection against oxidative stress. This study provides the first thorough description of parotid peroxisomes in different parotid cell types of healthy human tissue.

## 1. Introduction

Saliva plays an essential role in mastication, speech, protection, deglutition, digestion, excretion, and tissue repair. Salivary glands are exocrine glands that produce and secrete saliva using a system of ducts and acini. Humans have about 800–1000 minor salivary glands and three major paired salivary glands: parotid glands, sublingual glands, and submandibular glands. Of these, the parotid can be described as the largest, bordered anteriorly and medially by the masseter, superiorly by the zygomatic arch, and posteriorly by the sternocleidomastoid. This gland produces a serous fluid that helps with swallowing, chewing, digestion, and phonation [1]. The produced serous secretion comprises rich amylase, sialomucins, sulfomucins, ions, and water along with glycoconjugates that bind to calcium and are responsible for antimicrobial and enzymatic activities in saliva [2]. As the parotid gland has high intrinsic RNase activity, it is particularly challenging to extract intact RNA. We compared different methods to extract intact RNA from murine and human parotid gland tissue without losing the RNA quality [3].

All eukaryotic cells except erythrocytes and spermatozoa include the single membrane-bound organelle called a peroxisome [4]. The shape, size, quantity, and protein content of peroxisomes differ depending on the organism or cell type being studied [5]. The production of cholesterol and plasmalogens, as well as lipid metabolism, are closely related to peroxisomal functions [4]. Furthermore, peroxisomes play a crucial role in the processes of cellular signaling in inflammatory pathologies [5]. Most information on peroxisomes derives from lung, kidney, or liver research. As shown earlier by our workgroup, healthy and malignant tissue of the human parotid salivary gland express peroxisomal proteins differently. The fact that biosynthesis was upregulated while important antioxidant enzymes were downregulated showed that peroxisomes play a pro-tumorigenic role in parotid gland cancers [6]. However, to the best of our knowledge, minimal information is available on the biology of peroxisomes in the different cell types of human parotid glands.

The first series of experiments and research on peroxisomes in the human parotid gland were provided by Riva et al. in the late 90s. The authors exploited the power of electron microscopy using the DAB method and showed the cytochemical localization of catalase [7,8]. Subsequently, peroxisomes in rat parotid glands were defined by utilizing an improved DAB method by Graham and Karnovsky [9,10]. The authors that used this improved method observed the sporadic existence of peroxisomes in intercalated duct cells and acinar cells; however, they concluded that the peroxisomes were more frequent in the striated duct cells [9]. Based on these studies, they presented the detailed ultrastructure of excretory ducts in the parotid glands of rats and defined the occurrence of peroxisomes in epithelial cells [11]. Meanwhile, the existence of peroxisomes in the murine parotid gland was confirmed by employing Karnovsky’s DAB method for determining catalase distribution [12]. Tandler and Walter later used a novel method to confirm the existence of peroxisomes in the parotid glands of free-tailed bats [13,14,15].

However, there is still a considerable amount of work to be done in targeting the localization and characterization of peroxisomal proteins. Therefore, this study aimed to characterize and localize peroxisomal proteins and enzymes in the acinar and striated ducts cells of human parotid glands by employing electron- and light-based microscopic techniques combined with molecular analyses.

## 2. Results

Peroxisomes are numerous, and their protein content is highly abundant in the human parotid glands; however, there were significant cell-specific differences observed in their numerical abundance and enzyme content in the acinar and striated duct cells. 

**Figure 1:**
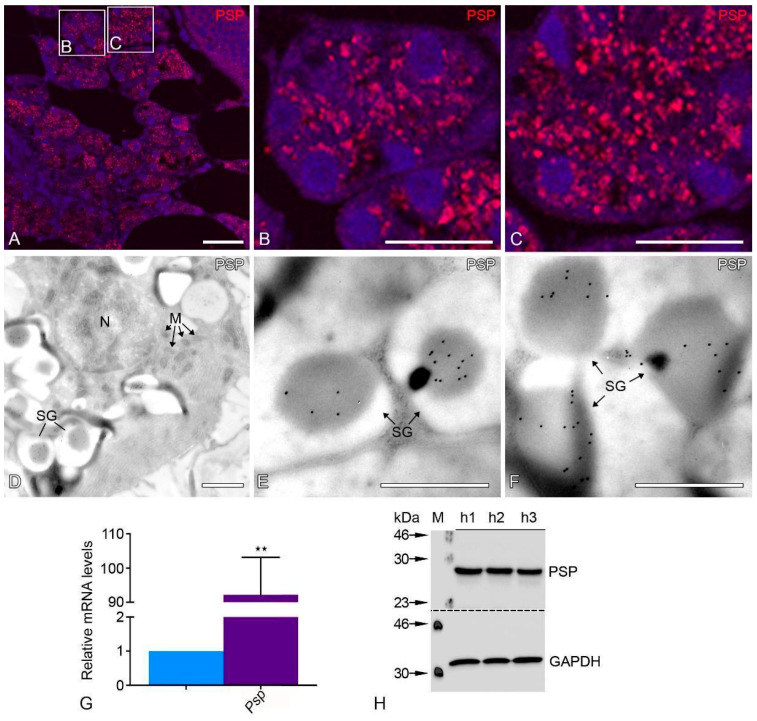
PSP staining is used to identify human parotid gland tissue. Human parotid gland immunofluorescence labeling for PSP in certain areas (**A**) and higher magnification PSP staining in human acini (**B**) and human striated ducts (**C**). Ultrastructural immunocytochemical localization of PSP staining in secretory granules of the human parotid gland (**D**) using the ultra-small gold (10 nm) method on LR White sections with post-embedding protein immunocytochemistry. Higher magnification view of a region containing PSP positive secretory granules of human acini (**E**,**F**). N = nucleus, M = mitochondria, SG = secretory granules. Bars represent: 50 µm (**A**); 10 µm (**B**,**C**); 5 µm (**D**) 2 µm (**E**,**F**). qRT-PCR analyses on human parotid tissues showing mRNA levels encoding for parotid specific protein (violet bar) compared to *Gapdh* (blue bar) as a housekeeping gene (** *p* < 0.0011) (**G**). PSP and GAPDH were probed on Western blots using an affinity-purified antibody (**H**).

Parotid tissue was identified with parotid specific protein (PSP) staining. Since the human parotid gland was surgically removed, we ascertained the origin of the isolated tissue with regular morphology before labeling it with antibodies against proteins that are located inside peroxisomes. The human parotid gland tissue showed the typical anatomical structure of lobes and lobuli with intralobular adipose tissue (Figure 1A–C) and exhibited a gland structure of pure serous acini (Figure 1B). The duct system consists of intercalated ducts, striated ducts, excretory ducts, and main excretory ducts (Figure 1C). As already described in the literature, PSP binds to the membrane of secretory granules and is therefore suitable for detecting parotid tissue. The parotid tissue, which was used for further experiments, reacted clearly positive to PSP staining (Figure 1A–C) [16]. Images of the tissue at a higher magnification show serous secretory cells and striated duct cells with several large and plentiful secretory granules (Figure 1B,C). We subsequently utilized post-embedding immunocytochemistry and the ultra-small gold technique at electron microscopic levels to examine the subcellular localization of PSP. Figure 1D–F demonstrates that gold particles are selectively detected in the secretory granules, indicating that the PSP antibody is highly specific. Organelles of other cells, including mitochondria, were negative. The results of the *Psp* mRNA expression analysis supported the morphological findings. When compared to *Gapdh* in Figure 1G, the *Psp* mRNA expression in the human parotid gland was substantially higher. Western blot analysis for PSP yielded a specific band at 28 kDa, as seen in Figure 1H. These results suggest that PSP is an exclusive parotid-specific marker protein and is highly abundant in secretory granules.

### 2.1. Peroxisomes Are Highly Abundant in the Human Parotid Gland

**Figure 2:**
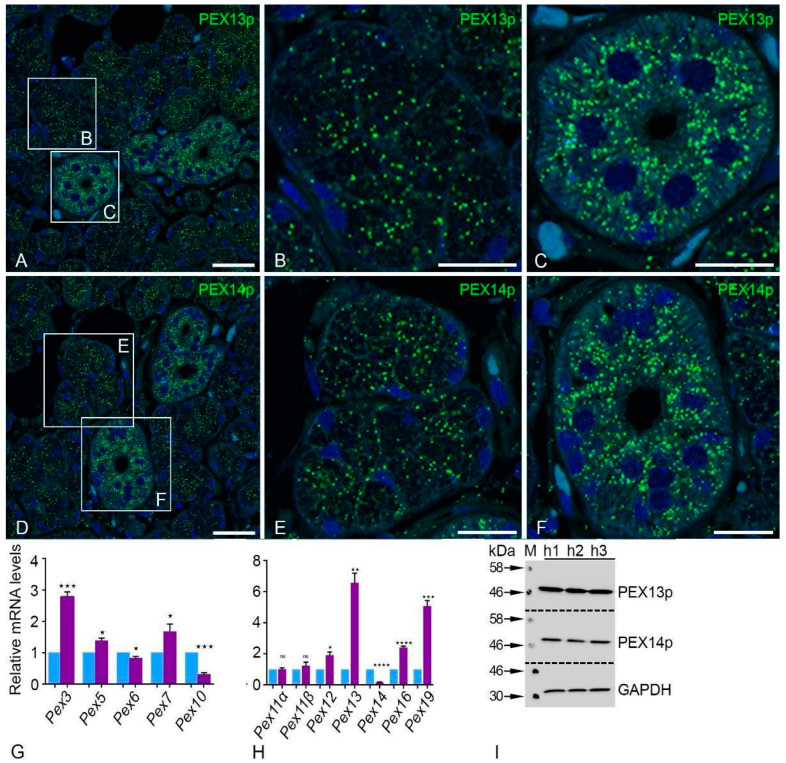
Employing antibodies against PEX13p and PEX14p and visualization with fluorescent secondary antibodies demonstrated high abundance of peroxisomes in the human parotid glands (**A**–**F**). Bars represent: 100 µm (**A**,**D**); 50 µm (**B**,**C**,**E**,**F**). qRT-PCR analyses on human parotid tissues showing mRNA levels encoding for peroxisomal biogenesis proteins (violet bar) compared to *Gapdh* (blue bar) as a housekeeping gene (**G**,**H**). For the following mRNAs, the respective *p*-values are *Pex3* *** *p* < 0.0004, *Pex5* * *p* < 0.0102, *Pex6* * *p* < 0.0376, *Pex7* * *p* < 0.0468, *Pex10* *** *p* < 0.0002, *Pex12* * *p* < 0.0111, *Pex13* ** *p* < 0.0008, *Pex14* **** *p* < 0.0001, *Pex16* **** *p* < 0.0001, *Pex19* *** *p* < 0.0003, ns: not significant. PEX13p and PEX14p were used as probes in a Western blot analysis to measure their expression. GAPDH was employed as a loading control (**I**).

We determined the peroxisomal compartment’s distribution pattern in the human parotid gland using peroxisome-specific antibodies. Interestingly, the peroxisomes in the human parotid gland are highly abundant (Figure 2A–F). Immunofluorescence analyses for PEX13p and PEX14p showed a punctate pattern that is typical of peroxisome staining. However, clear visible differences were observed between the acinar cells (Figure 2B,E) and striated duct cells (Figure 2C,F). Acinar cells displayed smaller amounts of stained peroxisomal proteins, as shown by labeling with PEX13p and PEX14p, compared to the striated duct cells (Figure 2B,C,E,F). Both proteins were strongly labeled with peroxisomes in the striated ducts (Figure 2C–F). In light of this, PEX13p and PEX14p Western blot analysis supported the morphological findings, indicating the abundance of both proteins in the human parotid gland (Figure 2I). Further, qRT-PCR analysis for most mRNAs coding for peroxisomal biogenesis proteins (*Pex3*, *Pex5*, *Pex7*, *Pex12*, *Pex13*, *Pex16*, *Pex19*) and peroxisomal proliferation proteins (*Pex11α*, *Pex11β*) revealed significantly higher expression levels compared to *Gapdh* (all *p* < 0.0128) in human parotid glands. In contrast, *Pex6*, *Pex10*, and *Pex14* showed lower expression levels than *Gapdh* (Figure 2G,H).

### 2.2. Peroxisomal β-Oxidation Enzymes Are Expressed at High Levels in the Human Parotid Gland

**Figure 3:**
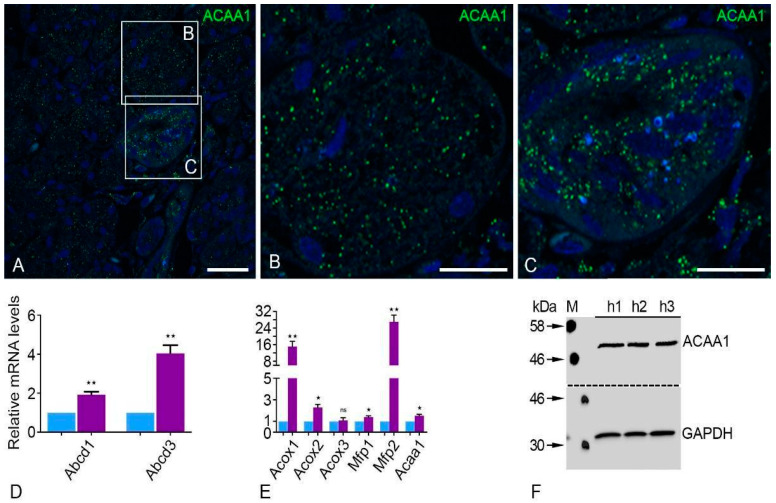
mRNAs encoding for proteins localized to peroxisomes and the corresponding proteins involved in the pathway for oxidizing and transporting lipids. Employing antibodies against and visualization with fluorescent secondary antibodies demonstrated high abundance of peroxisomes in the human parotid glands (**A**–**F**). IF staining showed peroxisomal ß-oxidation enzyme 3-oxo-acyl-CoA thiolase was abundant in the human parotid gland (**A**) and corresponding higher magnification of human acini (**B**) and striated ducts (**C**). Bars represent: 100 µm (**A**); 50 µm (**B**,**C**). qRT-PCR analyses for mRNAs encoding peroxisomal lipid transporters and enzymes involved in the peroxisomal ß-oxidation pathways (violet bars) were highly expressed in human parotid glands compared to *Gapdh* (blue bars) (**D**,**E**). qRT-PCR analyses on human parotid tissues showing mRNA levels encoding for β-oxidation enzymes compared to *Gapdh* as a housekeeping gene had the following *p*-values: *Abcd1* ** *p* < 0.0034, *Abcd3* ** *p* < 0.0020, *Acox1* ** *p* < 0.0050, *Acox2* * *p* < 0.0113, *Mfp1* * *p* < 0.0265, *Mfp2* ** *p* < 0.0014, *Acaa1* * *p* < 0.0134, ns: not significant. Western blot analysis of thiolase expression (**F**). GAPDH was used as a loading control.

We also investigated the peroxisomal β-oxidation enzymes in the human parotid gland. In particular, peroxisomal thiolase (ACAA1), which is involved in the last reaction of peroxisomal β-oxidation, demonstrated a typical peroxisomal staining similar to the peroxisomal biogenesis proteins (Figure 3A–C). Striated duct cells showed more intense labeling of peroxisomal proteins than acinar cells (Figure 3A–C). The qRT-PCR analysis of mRNAs for lipid transporters of the distinct ATP binding cassette subfamily D (*Abcd1* and *Abcd3*) also showed significantly higher expression levels in the human parotid gland in comparison to *Gapdh* (Figure 3D). Furthermore, the human parotid gland showed significantly higher expression of all β-oxidation enzymes (*Acox1*, *Acox2*, *Mfp1*, *Mfp2*, and *Acaa1*) except for Acyl-CoA oxidase 3 (*Acox3*), which was not expressed at a significantly higher level compared to *Gapdh* (Figure 3E). Of all the peroxisomal β-oxidation enzymes tested, the mRNA encoding for the protein *Mfp2* showed the highest expression in comparison to *Gapdh* (Figure 3E). Western blot examination confirmed the IF and qRT-PCR findings by demonstrating the presence of peroxisomal thiolase (ACAA1) in the human parotid gland (Figure 3F).

### 2.3. Plasmalogen Synthesizing Enzymes Are Expressed at Significantly High Levels in Human Parotid Glands

**Figure 4:**
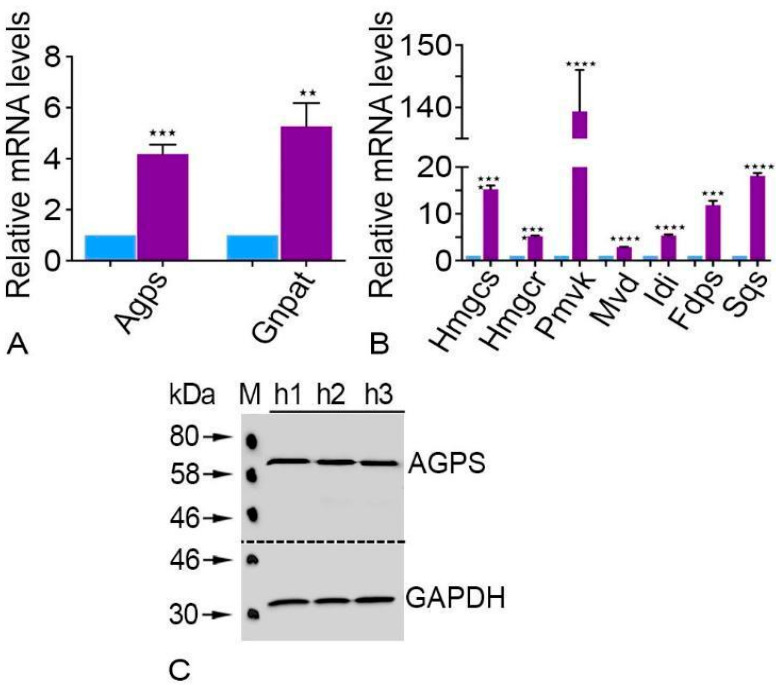
Peroxisomal ether lipid and cholesterol synthesis in human parotid gland tissues. qRT-PCR analyses for mRNAs encoding peroxisomal ether lipid synthesizing enzymes (**A**) as well as cholesterol synthesizing enzymes (**B**) in human parotid tissues (violet bars) compared to *Gapdh* (blue bars) as a housekeeping gene. For the following mRNAs, the respective *p*-value is *Agps* *** *p* < 0.0010, *Gnpat* ** *p* < 0.0094, *Hmgcs* **** *p* < 0.0001, *Hmgcr* **** *p* < 0.0001, *Pmvk* **** *p* < 0.0001, *Mvd* **** *p* < 0.0001, *Idi* **** *p* < 0.0001, *Fdps* *** *p* < 0.0003, *Sqs* **** *p* < 0.0001. Western blot analysis for AGPS in human parotid gland tissues (**C**). GAPDH was used as a loading control.

Glycerone-phosphate O-acyl transferase (*Gnpat*) and alkylglycerone phosphate synthase (*Agps*) had significantly higher levels of expression in human parotid glands than *Gapdh*, according to qRT-PCR data (Figure 4A). Furthermore, compared to GAPDH, the AGPS Western blot analysis showed a considerably increased quantity of this plasmalogen-producing enzyme (Figure 4C).

### 2.4. Cholesterol Synthesizing Enzymes Were Expressed Significantly Higher in the Human Parotid Glands

All cholesterol synthesizing enzymes were expressed at high levels in the parotid gland (Figure 4B). The enzyme HMG-CoA reductase (*Hmgcr*), which is localized in both compartments (peroxisomes and endoplasmic reticulum), has considerably higher levels of mRNA expression in the human parotid gland than *Gapdh* does [16,17]. In addition, the expression of farnesyl diphosphate synthase (*Fsps*), phosphomevalonate kinase (*Pmvk*), and mevalonate 5-disphosphate decarboxylase (*Mvd*), which are also found in peroxisomes, were also expressed noticeably higher in contrast to *Gapdh* (Figure 4B). It was also shown that the human parotid gland has elevated levels of 3-hydroxy-3-methylglutaryl-CoA synthase (*Hmgcs*) and iso-pentenyl diphosphate isomerase (*Idi*). Human parotid gland expression of the mRNA encoding the ER enzyme squalene synthase (*Sqs*) was similarly found to be substantially higher than that of *Gapdh*.

### 2.5. Peroxisomal Antioxidative Enzymes Were Detected in the Human Parotid Gland

**Figure 5:**
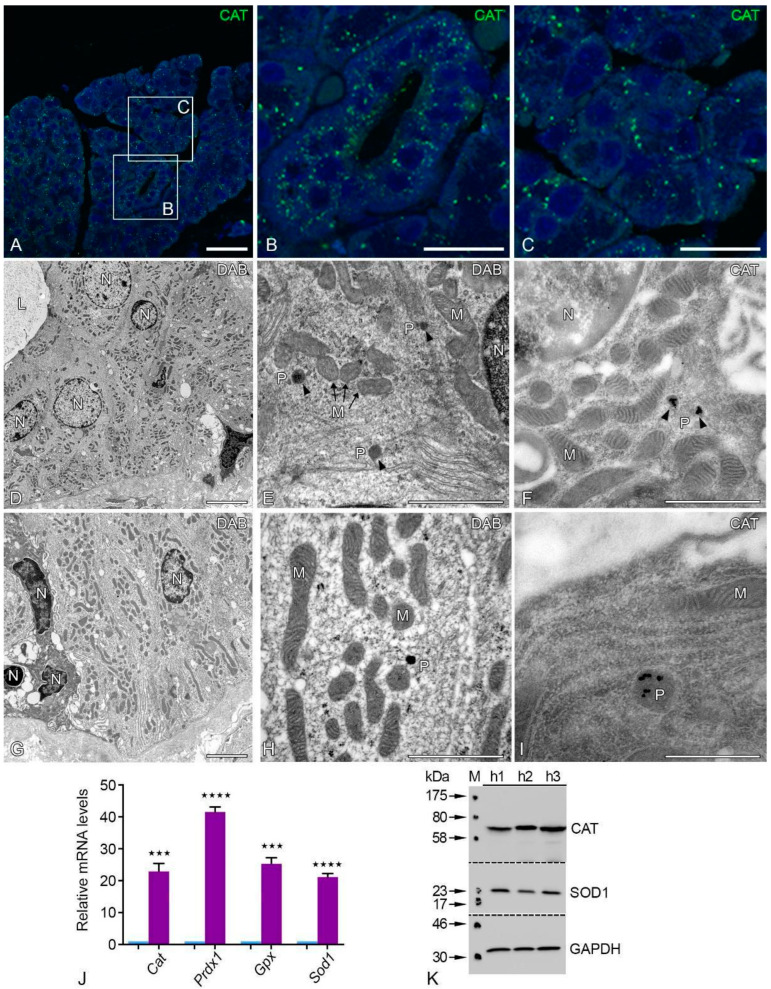
Ultrastructural catalase activity and protein localization in various cell types of the human parotid gland. IF staining of peroxisomal marker protein catalase in the human parotid gland (**A**) and the corresponding higher magnification of human acini (**B**) and striated ducts (**C**). Catalase activity is stained cytochemically using the modified alkaline DAB technique (**D**–**I**). Lower magnification view of an acinar region of the human parotid gland (**D**). Respective higher magnification view of human acinar cell with a peroxisome adjacent to several mitochondria I. Similarly, lower magnification view of human parotid gland striated duct epithelial cells (**G**). Respective higher magnification view of a striated duct cell with a peroxisome adjacent to several mitochondria (**H**). The catalase protein was located using electron microscopy, immunocytochemistry, and the protein A-gold method after embedding slices of human parotid glands on LR white (**F**,**I**). View of an area in a human acinar cell under higher magnification that contains peroxisomes and mitochondria (**E**,**F**). Similarly, a higher magnification view of striated ducts containing peroxisomes (**H**,**I**). Arrowheads depict peroxisomes; N: nucleus; M: mitochondria; P: peroxisome, L: lumen. Bars represent: 100 µm (**A**); 50 µm (**B**,**C**); 10 µm (**D**,**G**); 5 µm (**E**,**F**); 0.25 µm (**H**); 0.5 µm (**I**). qRT-PCR analyses for mRNAs encoding peroxisomal antioxidative enzymes in human parotid tissues (violet bars) compared to *Gapdh* (blue bars) as a housekeeping gene (**J**). For the following mRNAs the respective *p*-values are *Cat* *** *p* < 0.0009, *Prdx1* **** *p* < 0.0001, *Gpx* *** *p* < 0.0002, *Sod1* **** *p* < 0.0001. Western blot analysis for CAT and SOD1expression in human parotid gland tissues (**K**). GAPDH was used as a loading control.

Peroxisomal catalase staining revealed the typical punctate distribution with numerous large peroxisomes in acinar and striated duct cells of the human parotid gland (Figure 5A–C). We used a modified protocol based on the alkaline DAB method, which allowed us to detect the peroxisomes in the acinar and striated duct cells of the human parotid gland (Figure 5D–I) [18]. The ultrastructure of acinus cells showed a well-developed rough endoplasmic reticulum (rER), mitochondria, and nuclei with euchromatin. The mitochondria were in physical closeness to the nucleus and rER (Figure 5D–H). Peroxisomes were often closely associated with mitochondria and rER (Figure 5E,F,H). We investigated the localization of the catalase protein using post-embedding immunocytochemistry with ultra-small nanogold in addition to the localization of catalase activity at the electron microscopic level. Ultra-small nanogold particles were only found in the peroxisomal matrix of human acinar and striated duct cells, as illustrated in Figure 5I. The nuclei, mitochondria, and other cell organelles were not labeled. Negative controls using the PAG or ultra-nano gold technique on LR white sections revealed relatively few randomly arranged nanogold particles.

The peroxisomes had a round shape and showed the typical single membrane-bound border with a distribution pattern next to mitochondria and the nucleus (Figure 5E,F,H,I). The parotid glands also contain antioxidative enzymes from various subcellular compartments in addition to catalase to defend against oxidative damage. In comparison to *Gapdh*, the human parotid gland revealed a significantly increased expression of mRNAs encoding for peroxisomal antioxidative enzymes, such as peroxiredoxin 1 (*Prdx1*), glutathione peroxidase (*Gpx*), and superoxide dismutase 1 (*14*). The Western blot analysis for CAT and SOD1 revealed the abundance of these peroxisomal antioxidative enzymes in the human parotid gland (Figure 5K).

### 2.6. Antioxidative Enzymes of Different Cell Compartments Were Also Abundant in Human Parotid Glands

**Figure 6:**
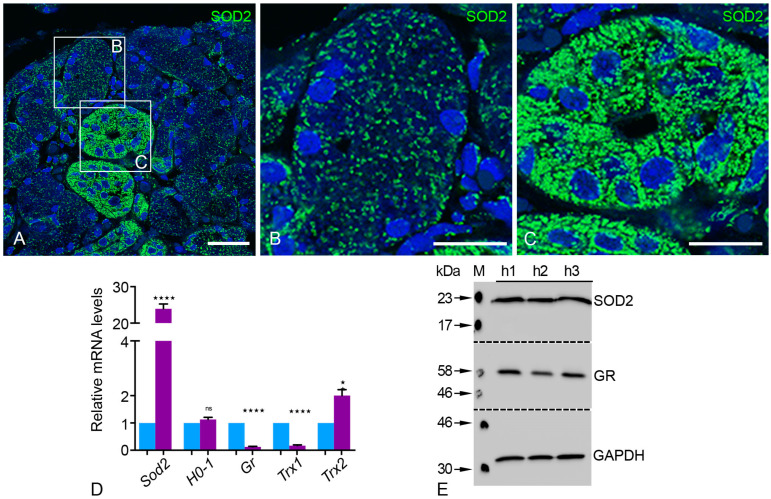
The antioxidant enzymes of different cell compartments in human parotid gland tissue. IF staining of SOD2 in the mitochondria showed the abundance in human parotid gland tissue (**A**) and enlarged sections of acini (**B**) and striated ducts (**C**). Bars represent: 100 µm (**A**); 50 µm (**B**,**C**). qRT-PCR analyses of mRNAs encoding for antioxidative enzymes of different cell compartments in human parotid glands (violet bars) compared to *Gapdh* (blue bars) as a housekeeping gene (**D**). For the following mRNAs the respective *p*-value is *Sod2* **** *p* < 0.0001, *Gr* **** *p* < 0.0001, *Trx1* **** *p* < 0.0001, *Trx2* * *p* < 0.0097, ns: not significant. Western blot analysis for SOD2 and GR expression in human parotid gland tissue (**E**). GAPDH was used as a loading control.

**Figure 7:**
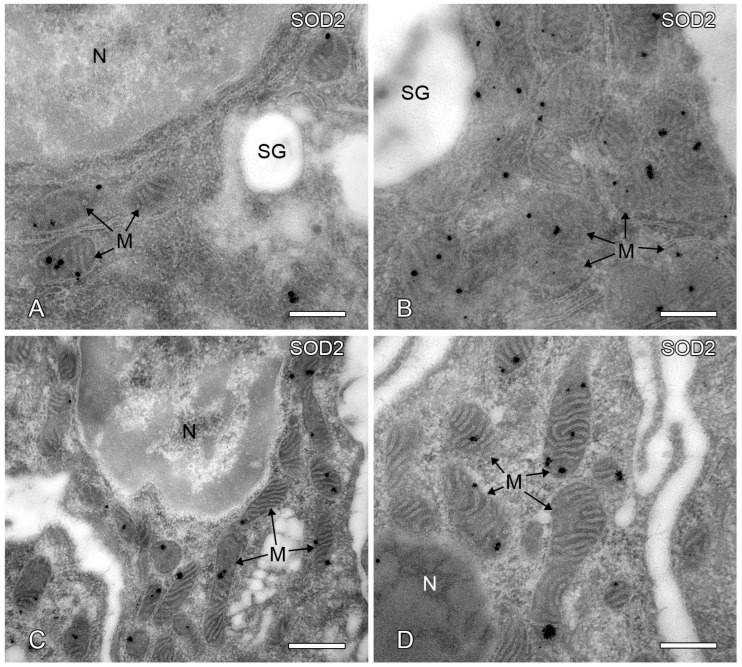
Ultrastructural immunocytochemical localization of SOD2 in human parotid gland tissues. Electron microscopic immunocytochemical localization of the SOD2 protein in human parotid gland acinar (**A**,**B**) and striated duct cells (**C**,**D**) in the ultrathin sections with ultra-small nanogold on LR white sections. Arrows depict mitochondria; SG: secretory granules; N: nucleus; M: mitochondria. Bars represent: 1 µm (**A**,**C**); 0.5 µm (**B**,**D**).

The mitochondrial superoxide dismutase 2 (SOD2) was detected via immunofluorescence staining and showed a typical localization pattern of the mitochondria in the acinar and striated duct cells (Figure 6A–C). We found clear and robust differences in the number, shape, and morphology of mitochondria between the acini and the striated ducts of human parotid glands. The SOD2 staining showed that the mitochondria were less numerous and displayed a round pattern in the acini compared to the more numerous and elongated form in striated duct cells (Figure 6A–C and Figure 7A–D). Most SOD2-labeled mitochondria were detected in the basal portion of the striated duct epithelial cells and significantly less at the lateral sides and apical portion of the cells (not shown). Strong SOD2 labeling, on the other hand, revealed elongated and tubular mitochondria with extensive network formation throughout the striated duct cells of the parotid gland (not shown). SOD2 is a crucial superoxide radical scavenger that transforms superoxide radicals into less harmful H_2_O_2_ in the mitochondrial matrix (Figure 6C).

Interestingly, the qRT-PCR analysis of mRNAs encoding for *Sod2* and *Trx2* showed a significantly higher expression than *Gapdh* (Figure 6D). The distribution of different thioredoxin isoenzymes suggests that human parotid gland cells also appear to contain a specialized set of antioxidant enzymes in addition to *Sod2*. *Trx2* was more highly expressed in comparison to *Trx1* and glutathione reductase (*Gr*) in the human parotid gland (Figure 6D). Western blot analysis of antioxidative enzymes showed the abundance of SOD2 and GR in agreement with the qRT-PCR analyses (Figure 6E).

### 2.7. Post-Embedding Immunoelectron Microscopy of SOD2 Localization

We chose to investigate the subcellular localization of the SOD2 protein by post-embedding immunocytochemistry of LR white ultrathin cryosections using ultra-small gold-labeled Fab fragments and silver intensification as a secondary detection method in order to achieve the highest sensitivity labeling for SOD2. Our results showed that the ultra-small gold particles used to visualize SOD2 were explicitly and exclusively confined to mitochondria in human acinar and striated duct cells (Figure 7A–D). In immunostainings using SOD2 antigen-specific antibodies, we did not find any gold particles in any other cell compartments. 

### 2.8. Peroxisome Proliferator-Activated Receptors (PPARs) Are Highly Expressed in the Parotid Gland

**Figure 8:**
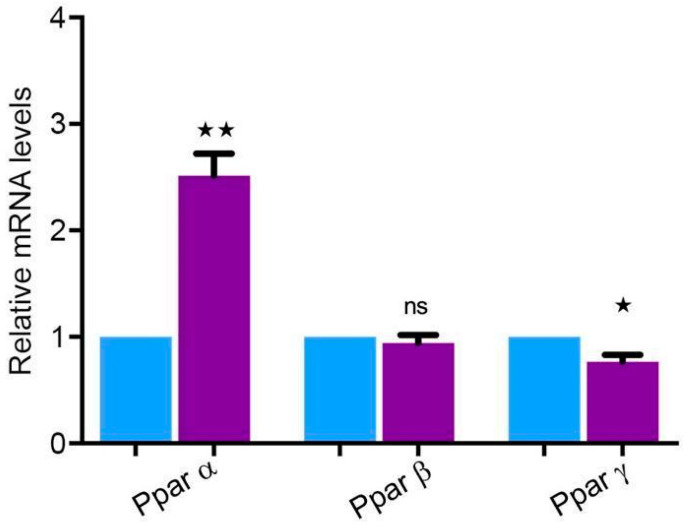
Peroxisomal proliferator-activated receptors in human parotid gland tissue. qRT-PCR analyses for mRNAs encoding for peroxisomal proliferator-activated receptors in human parotid tissues (violet bars) compared to *Gapdh* (blue bars) as a housekeeping gene. *Pparα* was highly expressed in human parotid glands, whereas the expression of mRNA encoding for *Pparβ* showed no statistical significance. *Pparγ* showed decreased expression. For the following mRNAs the respective *p*-value is *Pparα* ** *p* < 0.0018, *Pparγ* * *p* < 0.0213, ns: not significant.

It is well known that several peroxisomal proteins involved in lipid metabolism and oxidative stress and the genes encoding for them are regulated by the peroxisome proliferator-activated receptors (PPARs). There are three family members of the PPARs: *Pparα*, *Pparβ*, and *Pparγ*. *Pparα* was expressed significantly higher in the human parotid gland, whereas *Pparγ* was expressed significantly lower compared to *Gapdh*. Furthermore, the expression level of *Pparβ* did not show any significant differences compared to *Gapdh* (Figure 8).

## 3. Discussion

The parotid gland is an organ that has an important role in the immune and digestive systems. Salivary glands secrete the necessary proteins that initiate the digestion process and provide tissue lubrication in the oral cavity, and they play a vital role in fighting infections and oxidative stress [19,20,21,22]. A plethora of work has been conducted on the cell biology of the parotid gland and the proteome of saliva [23,24,25]. Recent studies have shown that oxidative stress accompanies parotid gland tumors, suggesting that it plays an important part in the pathogenesis of parotid gland tumors [21]. Peroxisomes harbor a set of antioxidative enzymes, and they are a vital player in the degradation of nitrogen and reactive oxygen species [5]. However, to the best of our knowledge, no significant work is available yet that explains the potential role of peroxisomes and their distribution in different cell types of healthy human parotid glands. Therefore, we explored the role of antioxidative enzymes, peroxisomes, and metabolizing enzymes in the different subcellular sections by using light-, electron-, and immunofluorescence microscopy. 

The results confirmed the presence of peroxisomes in all cell types of the human parotid gland. It was also shown that there is a substantial difference in the abundance of peroxisomal proteins in acinar cells compared to striated duct cells. Human parotid glands contain high quantities of catalase and other antioxidative enzymes in distinct subcellular sections as well as mRNAs encoding for multifunctional protein 2 and Acyl-CoA oxidase 1.

### 3.1. Marker Proteins for the Correct Identification of the Parotid Gland

We utilized PSP to categorize the isolated tissue and detected this protein in the parotid gland by using post-embedding immunocytochemistry. The patterns of PSP staining and the respective protein expressions are equivalent to the data provided by Bingle et al., which confirms that parotid tissue was isolated [26]. Moreover, the authors have shown that PSP is an excellent marker to distinguish parotid tissue from surrounding tissue due to its different expression patterns in various tissues and glands [26]. Despite this, the exact role and abundance of PSP is still unknown [26].

### 3.2. Peroxisomes in the Parotid Gland

Electron microscopy was initially used to identify peroxisomes utilizing the cytochemical localization of catalase activity in the parotid glands of humans, mice, and rats [7,8,9,11,12]. These works confirmed the higher number of peroxisomes in the excretory and striated ducts of parotid glands. Few peroxisomes were found in the cells of intercalated and acinar ducts. Grant et al. showed immunofluorescence staining of PEX14p in human submandibular glands [27]. The location of peroxisomal enzymes and the gene expression-based profile of the proteins involved in peroxisomal biogenesis were not specifically covered in the literature. The best peroxisomal generator protein, PEX14p, is evenly distributed throughout the parotid gland [4,28,29]. In addition to PEX14p, the human parotid gland has many metabolic and peroxisomal biogenesis proteins, as well as antioxidative enzymes. This confirms the importance of peroxisomes in lipid metabolism and their role in the reduction of oxidative stress. The mRNAs encoding the PEX11α, -β, and -γ proteins that are involved in the peroxisomal proliferation are also present in the human parotid gland [30,31,32]. The peroxisome count and the respective morphological structure rely on metabolic need and its cell-specific functions [33,34].

### 3.3. In the Human Parotid Gland, Peroxisomal ß-Oxidation, Cholesterol Production, and Plasmalogen Synthesis Enzymes Are Highly Expressed

Degradation of bioactive lipids is assisted by peroxisomal β-oxidation. Eicosanoids, for instance, play a role in the production of polyunsaturated fatty acids and the process of inflammation [35]. The prevalence of peroxisomal thiolase in striated duct and acinar cells must be discussed. The rate-limiting enzymes of pathway 1 of the peroxisomal β-oxidation are peroxisomal enzymes, such as acyl-CoA oxidase 1–3 (ACOX). This helps to regulate the substrate flux by using the β-oxidation chain [36]. The human parotid gland has strongly expressed mRNAs for the distinct peroxisomal β-oxidation pathway 1 (*Mfp1*) and peroxisomal β-oxidation pathway 2 (*Mfp2*). The metabolism of straight-chain substrates is the primary focus of the MFP1 enzyme, but MFP2 regulates a sizable portion of the substrates for peroxisomal β-oxidation [37]. It is also worth mentioning that the abundance of such enzymes can guard the epithelium against proinflammatory eicosanoids. The high involvement of ABCD3 in the human parotid gland shows that the transporter facilitates an ingress of the branch-like long-chain unsaturated and saturated substrate into the peroxisomes [38]. However, the precise function of peroxisomal β-oxidation in the lipid-transport and homeostasis has not yet been investigated.

Peroxisomal β-oxidation can provide the acetyl-CoA units to generate lipids such as plasmalogens or cholesterol precursors [39]. The lipid synthesizing enzymes present in the peroxisomes might play a vital role in the parotid gland. For example, AGPS, which is abundant in the parotid gland, is involved in the synthesis of ether lipids. Ether lipids are known to trap the reactive oxygen species (ROS) (Karnati and Baumgart-Vogt, 2008); therefore, peroxisomes in the parotid gland might help against oxidative damage. Plasmalogens, the largest class of ether lipids, promote the formation of biologically active lipids for cellular signaling [40]. Remarkably, the abundance of lysoplasmalogens is directly linked with the electrophysiological instabilities in myocytes that repress Na^+^–K^+^-ATPase in the renal cells [41]. The striated duct cells contain Na^+^–K^+^-ATPase in the basolateral and lateral part of the epithelial cells; however, the function of plasmalogens in striated duct cells is so far unidentified. 

Cholesterol is also a crucial lipid and a mandatory element of bile acids, steroid hormones, and oxysterols [42]. Like other peripheral tissues, the parotid gland uses cholesterol for cellular growth, as was found in rats [43] and mice [44]. It is worth noting that the peroxisomal enzymes can condense acetyl-CoA, which is derived from long-chain fatty acid oxidation, into farnesyl diphosphate (FPP). The reactions of FPP and mevalonate are exclusively peroxisomal except for the reaction of HMG-CoA reductase [45]. The mRNAs of all proteins involved in the synthesis of cholesterol are found to be abundant in the parotid glands of humans, which can affect cholesterol metabolism. In fact, diabetes mellitus and parotid cholesterol metabolism are linked, as evidenced by the discovery of asymptomatic parotid gland enlargement in diabetic rats [46]. More research is needed to determine how low insulin levels specifically affect parotid cholesterol metabolism.

High concentrations of antioxidative enzymes can be found in several subcellular compartments of the human parotid gland. Saliva is comprised of antioxidants with special characteristics to protect against oxidative stress [22]. Previous observations have also highlighted that hyposalivation produces oxidative stress by harming the salivary gland’s structure [47,48]. In this respect, it is essential to highlight that the parotid gland possesses peroxisomal enzymes and several other antioxidative catalysts that detoxify H_2_O_2_ produced by peroxisomal oxidases [28]. The striated duct cells within the human parotid gland possess an excessive amount of mitochondrial SOD2, an essential scavenging catalyst that transforms superoxide radicles created by the mitochondrial respiratory chain into less toxic H_2_O_2_. Several studies have already shown that ROS derived from mitochondrial production grows with age whereas the body’s antioxidative potential decreases [49]. Therefore, the accumulation of ROS becomes harmful for phospholipids and the cell membrane, which is the primary cause of cellular dysfunction [50]. It is worth mentioning that the human parotid gland possesses antioxidant enzymes that are cleared from the thioredoxin isoenzyme distribution. Intriguingly, mRNAs encoding TRX1, certain PEX genes, and cytoplasmic GR were expressed significantly less in parotid gland tissue. It seems that decreasing the antioxidative potential of saliva can increase the vulnerability of the salivary gland to oxidative destruction and maximize the oxidative stress that is related to oral maladies (dental caries, burning mouth syndrome, and oral inflammatory infections like gingivitis, periodontitis, oral mucosa ulceration, and candidiasis) [51].

### 3.4. PPARs Are Expressed at a Significantly High Level in the Human Parotid Gland

PPARs play vital roles in glucose metabolism, lipid metabolism, aging, stress, and producing transcription factors [52,53,54]. PPARα is highly expressed within the parotid gland of humans, which may explain the peroxisomal compartment induction. It has already been shown that PPARγ also regulates genes for PEX11, a large number of peroxisomal β-oxidation enzymes, and ABCD transporters by attaching to the PPARs’ responsive regions (PPRE) [34,54,55]. Most of the research on the PPARs’ role in parotid tumors has just recently been published. PPARγ upgrades Sjögren’s syndrome, the over-expressive regulation of cytokines within the peripheral blood or salivary gland, in non-obese diabetic mice [56]. PPARα and PPARγ could inhibit IL-1β-made NO growth in cultured cells of the lacrimal gland acini, proposing that PPARs might be a beneficial therapy target for avoiding NO-mediated gland destruction. Despite this, the PPARα and PPARγ effects on the development of salivary gland dysfunction are not apparent.

## 4. Materials & Methods

### 4.1. Surgical Removal and Fixation of the Human Parotid Glands

The human parotid tissue was removed during surgical operations on benign parotid gland tumors following the standard operating procedures. Pathologists examined the samples at the Institute for Pathology of the Justus Liebig University Giessen, Germany. Informed patient consent was obtained from all individual participants included in this study. We collected tissue samples during surgery performed on the parotid gland. The obtained tissue was divided. One part was fixed with 4% PFA in PIPES buffer with 2% saccharose and 0.05% glutaraldehyde at pH 7.4 for electron microscopy, another part was snap-frozen in liquid nitrogen for Western blotting, and the last part of the parotid tissue was either immersed in RNA later, with subsequent freezing, or immersed in 4% PFA in PBS at pH 7.4 and kept at 4 °C for paraffin embedding. The ethical review committee of the Justus–Liebig University Giessen approved removing and examining the human tissue (AZ 95/15, 25 June 2015). All procedures performed in studies involving human participants followed the ethical standards of the institutional and national research committee and the 1964 Helsinki declaration and its later amendments or comparable ethical standards.

### 4.2. Paraffin Embedding, Sectioning, and Immunofluorescence

It was previously documented in detail how the tissues were sectioned, paraffin embedded, and then used for antigen retrieval and immunofluorescence [4,28,29]. To read more about the primary and secondary antibodies used to incubate the sections with antibodies against peroxisomal, mitochondrial, and other proteins, see Table 1. In immunofluorescence preparations of paraffin slices of different tissues, all antibodies against peroxisomal proteins had already undergone testing for their specificities (lung [28], brain [57], and testes [58]). The sections were mounted in Mowiol 4.88 with N-propyl gallate in a 3:1 ratio after being counterstained with TOTO-3 iodide to detect nuclear morphology. Parallel negative controls incubated without primary antibodies. Using a Leica TCS SP5 confocal laser scanning microscope with a 63× objective and the “Airy 1” setting, the immunofluorescence preparations were analyzed.

### 4.3. Fixation and Embedding for Electron Microscopy

Fresh human tissue was placed into a 4% PFA fixative solution in a 0.1 M sodium cacodylate buffer with 2% sucrose at 4 °C. The embedding was performed with Epon or LR White following the manufacturer’s instructions and placed in a vacuum exsiccator to set. Afterward, the tissue was cut into sections using a thin razor blade. 

### 4.4. Cytochemical Localization of Catalase Activity with the Alkaline DAB Method

The cytochemical localization of catalase activity with the alkaline DAB-method in human parotid glands was performed as previously described [28]. Briefly, the human parotid gland slices were incubated with an alkaline DAB medium [18] containing 0.2% 3,3′-diaminobenzidine (DAB, Sigma, Steinheim, German), 0.15 % H_2_O_2_, and 0.01 M Teorell-Stenhagen buffer at pH 10.5. For the best catalase reaction, the reaction was conducted for two hours at 45 °C in a shaking water bath. The sections were then rinsed three times in 0.1 M cacodylate buffer with a pH of 7.4. The osmicated sections were dehydrated in a succession of increasing concentrations of ethanol solutions before being embedded in epoxy resin 812 (Agar, Essex, England). The trimming of the blocks was done with a diamond trimmer (Reichert TM 60, Austria) and the sectioning with a Leica Ultracut E Ultramicrotome (Leica, Nussloch, Germany). The ultrathin slides were collected on nickel grids covered with formvar, and contrasting was done using lead citrate for 45 s and uranyl acetate for two minutes. A transmission electron microscope, model LEO 906, was used for the study (LEO Electron Microscopy, Oberkochen, Germany).

### 4.5. Post-Embedding Immunoelectron Microscopy

According to Newman et al. [59], another portion of the previously fixed wet parotid sections was directly dehydrated after being exsiccated in 4% PFA-fixative and implanted in medium grade LR White resin (LR White Resin, Berkshire, England). As previously mentioned, slides were collected on formvar-coated nickel grids after ultrathin sectioning (80 nm) was completed. Blocking was performed with 1% bovine serum albumin (BSA) dissolved in a tris-buffered saline solution (TBS) at pH 7.4 for 30 min to prevent unspecific binding. The sections were treated with a rabbit anti-mouse catalase antibody (1:4000 in 0.1% BSA in TBS; a gift from Denis Crane, Table 2) overnight in a wet chamber. The following day, the sections underwent washing with drops of 0.1% BSA in TBS twelve times. The washed grids were incubated with a protein A-gold complex (PAG, gold particle size 15 nm) diluted with 0.1% BSA in TBS (1:75) [60]. The next step was distilled water washing, then air drying. Uranyl acetate was used for the contrasting for two minutes, followed by lead citrate for 45 s. Other ultrathin sections were used as the negative control, which were also treated with non-specific rabbit IgG and placed on grids. In contrast to the other sections, the negative control was followed by the protein A-gold complex alone without a primary antibody. Subsequently, a LEO 906 transmission electron microscope was used for the examination (LEO Electron Microscopy, Oberkochen, Germany).

### 4.6. Homogenization of Human Parotid Glands to Obtain Tissue Lysates for Western Blotting

Snap-frozen human parotid glands were cut into small pieces, and the tissue was homogenized in a buffer containing 0.25 M sucrose and 5 mM MOPS (pH 7.4), 1 mM EDTA, 0.1% ethanol, 0.2 mM DTT, 1 mM aminocaproic acid, and 100 µL cocktail of protease inhibitors (#39102, Serva, Germany). The tissue and homogenization buffer were used over an ice bath and treated with a single stroke of a Potter-Elvehjem homogenizer (B. Braun Biotech International, Melsungen, Germany) for 60 s at 1000 rpm. Centrifugation was done at 2500× g for 20 min at 4 °C to sediment connective tissue, nuclei, and giant mitochondria. The protein concentration was measured with the BCA Protein Assay Kit (Pierce, Thermo Fisher Scientific, Langenselbold, Germany) according to the manufacturer’s instructions using an Infinite M200 PRO NanoQuant plate reader (Tecan Group, Maennedorf, Switzerland) for measurement. 

### 4.7. Western Blot Analysis

The total proteins of the human parotid glands (40 µg) were separated on 10% resolving gels using the sodium dodecyl sulfate-polyacrylamide gel electrophoresis (SDS-PAGE). The SDS-PAGE was done using a Mini Protean Tetra electrophoresis module assembly and a Power Pac Basic (BioRad, Dreieich, Germany). Semi-dry blotting was used to transfer the proteins for 50 min at 10 V while utilizing a Trans-Blot Semi-Dry (BioRad, Dreieich, Germany) and a Protran nitrocellulose membrane (Whatman, Dassel, Germany). The membrane was blocked with 5% fat-free milk powder (Applichem, Darmstadt, Germany) in TBS plus 0.5% Tween 20 (Applichem, Darmstadt, Germany) (TBST) for 1 hr at RT. The primary antibodies were diluted in the blocking solution (exact dilution, see Table 1) and incubated overnight at 4 °C. After the incubation, washing of the membrane was performed with TBST (5 min) and TBS (2 × 5 min), and the secondary antibody (for dilution, see Table 1) was put on for 1 hr in RT diluted in 0.5% BSA in TBST. After the washing step, the detection of the immunoreactive bands was done using the Immun-Star WesternC Chemiluminescent Kit (BioRad, Dreieich, Germany) and the ChemiDoc XRS system (BioRad, Dreieich, Germany) for visualization. ImageLab Version 3.0 (BioRad, Dreieich, Germany) was used for image processing and analysis. The membranes were stripped with a 25 mM glycine and 10% SDS solution followed by a 100 mM sodium hydroxide and 10% SDS solution (15 min each) with subsequent reprobing. 

### 4.8. RNA Isolation

For RNA isolation, the fresh human tissue was immersed in RNAlater and snap-frozen in liquid nitrogen. The tissue samples were stored at −80 °C before further use. For homogenization of the tissue, a TissueLyser LT (Qiagen, Hilden, Germany) was used. Different methods for RNA isolation were already tested to achieve the best possible RNA quality [3]. Following the manufacturer’s instructions, the subsequent RNA isolation was carried out using the RNeasy Mini Kit (Qiagen, Hilden, Germany). The Agilent 2100 Bioanalyzer system and the Agilent RNA 6000 Nano Kit were used to confirm the RNA quality and concentration (Agilent Technologies, Santa Clara, CA, USA).

### 4.9. cDNA-Synthesis

The High-Capacity RNA-to-cDNA Kit (Applied Biosystems, Weiterstadt, Germany) was used for reverse transcription according to the manufacturer’s instructions together with a C1000 Thermal Cycler PCR system (BioRad, Dreieich, Germany).

### 4.10. Quantitative Reverse Transcriptase-Polymerase Chain Reaction (qRT-PCR)

The Primer Quest Tool (http://eu.idtdna.com/Primerquest/Home/Index accessed on 20 May 2020) was used to design the primers. Eurofins MWG Operon received the order for the primers. Table 2 contains a list of all the primers used. The StepOnePlus Real-Time PCR System (Life Technologies, Darmstadt, Germany) and SYBR Select Master Mix Kit (Life Technologies, Darmstadt, Germany) were used to perform qPCR in accordance with the manufacturer’s instructions. The PCR was conducted using a primer concentration of 5 pmol/L. Program used: 45 cycles of denaturation at 95 °C for 15 s, annealing at 60 °C for 60 s, and extension at 7 °C for 1 min.

### 4.11. Statistical Analysis

For the statistical analysis of the different mRNA expression levels, normalized values were used compared to a stable housekeeping reference gene. A Kolmogorov-Smirnov test was used for the normal distribution of the samples. The values are expressed as means ± SEM using the total RNA from human (n = 3) parotid gland samples. The difference in expression between the housekeeping gene and the target genes was evaluated using a Student’s *t*-test for unpaired samples. All statistical tests were calculated using the GraphPad Prism software version 6.01. 

## 5. Conclusions 

Our study’s findings showed that the staining of structures containing peroxisomal proteins is more intense in striated duct cells than in acinar cells, which may indicate that more of the corresponding proteins are present in striated duct cells. However, there is no evidence that the composition of peroxisomal proteins differs between the two cell types.

The human parotid gland exhibits a high number of peroxisomes and has distinct subcellular compartments with comparatively high concentrations of catalase and other antioxidative enzymes. This study strongly supports the idea that peroxisomal lipid metabolism also plays a crucial role in the parotid gland. Unraveling the precise metabolic and functional role of peroxisomes in the parotid gland should assist in understanding the cause of parotid tumors and guide the development of therapies.

## Figures and Tables

**Table 1:** List of antibodies used for the detection of peroxisomal proteins in the parotid gland.

Primary Antibody	Host	Target Molecular Weight	Dilution (IF)	Dilution (WB)	Supplier
Cell type specific antigens					
Parotid secretory Protein, human (SPLUNC2)	Mouse, monoclonal	25 kDa		1:1000	Abbexa, Cambridge, UK, Cat. no.: abx11413
Peroxisomal biogenesis and metabolic proteins					
Peroxin 13 (Pex13p), mouse	Rabbit, polyclonal	44 kDa	1:1000	1:6000	Gift from Denis I. Crane; School of Biomol. Biophys. Sci., Griffith Univ., Nathan, Brisbane, Australia
Peroxin 14 (Pex14p), mouse	Rabbit, polyclonal	42 kDa	1:1000	1:3000	Gift from Denis I. Crane
Catalase (CAT), mouse	Rabbit, polyclonal	60 kDa	1:2000	1:5000	Gift from Denis I. Crane
Thiolase	Rabbit, polyclonal	51 kDa	1:1000	1:5000	Gift from Nancy E Bravermann; Depts. of Human Genetics and Pediatrics, McGill University-Montreal Montreal, QC, Canada.
Alkylglycerone-phosphate synthase (AGPS)	Mouse, monoclonal	78 kDa	1:1000	1:500	Santa cruz, Cat no: sc-374201
Antioxidative enzymes from other cell compartments					
Gluthation reductase	Rabbit, polyclonal	56 kDa		1:1000	Abcam, Cambridge, UK, Cat. no: ab16801
Superoxide dismutase 1 (SOD-1),	Goat; polyclonal	17 kDa		1:5000	R&D Systems, Minneapolis, MN, USA, Cat. no.; AF3787
Superoxide dismutase 2 (SOD-2)	Rabbit, polyclonal	25 kDa	1:1000	1:1000	Research diagnostics, Inc., NJ, USA, Cat no: RDI-RTSODMabR
Other marker proteins of different cell compartments					
Glyceraldehyde-3-phosphate dehydrogenase (GAPDH)	Mouse, monoclonal	36 kDa		1:60,000	Hy Test Ltd., Cat. no.:5G4
Secondary Antibodies					
HRP-rabbit				1:6000	Cell Signaling Technology, Inc., Danvers, MA, USA
HRP-mouse				1:6000	Cell Signaling Technology, Inc., Danvers, MA, USA
Bovine anti goat HRP				1:5000	Santa cruz, Cat. no: sc-2378

**Table 2:** List of the human primers used for RT-PCR analyses.

Gene Target	Gene Bank Accession No.	Sence Primer (3′-3′)	Antisence Primer (5′-3′)	Annealing Temp °C	PCR Product (bp)
*ABCD1*	NM_000033.3	GTGGAGGACATGCAAAGGAA	TCACACATAGCCTCCCAACC	58.1	113
*ABCD3*	NM_002858.3	ATGACCCTTGGAACACTTCG	TGCCATCCATATGCAGGTAG	57.8	385
*ACOX1*	NM_004035.6	ATTTCCTTCAGGGGAGCATC	GCCAAGTGTCACATCCTGAA	57.3	137
*ACOX2*	NM_003500.3	CAAATTGTCGGCCTCCTGTA	GAGATCTCTGTGGCGTGGAG	57.9	125
*ACOX3*	NM_003501.2	GGAGTGTGTGGGCTCTTATC	CTCTTGCTCGGTAGGCATC	57.7	107
*ACTB*	NM_001101.3	TCCCTGGAGAAGAGCTACGA	AGCACTGTGTTGGCGTACAG	59.4	194
*AGPS*	NM_003659.3	AGGGGGATCGTGAGAAGGT	CCAAAGCCAAGTCTCGAATG	59.6	147
*CAT*	NM_001752.3	CGTGCTGAATGAGGAACAGA	TTGTCCAGAAGAGCCTGGAT	57.9	150
*FDPS*	NM_001135821.1	CAAGGAGGTCCTGGAGTACAA	GGAGACTATCAGCATCCTGTTTC	58.7	113
*GAPDH*	NM_002046.5	GTCAACGGATTTGGTCGTATT	TGTAGTTGAGGTCAATGAAGGG	56.6	106
*GNPAT*	NM_014236.3	GTGCAGAAAAACGCCTTAGC	GGCTGGTTTTCCTATTGGTG	58.3	150
*GPX1*	NM_000581.2	CAGTTTGGGCATCAGGAGAA	TCGAAGAGCATGAAGTTGGG	57.8	101
*GR1/GSR*	NM_000637.3	GTGGCCTCCTATGACTACCT	CATCCAACATTCACGCAAGTG	57.9	137
*HMGCR*	NM_000859.2	CGATGCATAGCCATCCTGTAT	GCTGGAATGACAGCTTCACA	57.7	87
*HMGCS*	NM_001098272.2	TCTATCCTTCACACAGCTCTTTC	GGCAACAATTCCCACATCTTT	57.9	89
*HO-1*	NM_002133.2	CGGCTTCAAGCTGGTGAT	AGCTCTTCTGGGAAGTAGACA	57.7	114
*HPRT*	NM_000194.2	CACTGGCAAAACAATGCAGACT	GTCTGGCTTATATCCAACACTTCGT	60.2	118
*IDI*	NM_004969.3	TCTCATTGGGCATGAAGGTC	CATAAAACCTCGGGCTCCTT	57.6	106
*MFP1*	NM_001966.3	ATGGATATGGATGGCCAAGG	GCTCCAGTTGGGGAATATCA	57.1	126
*MFP2*	NM_000414.3	TGTCGTTGCAGGCCTTATT	CCTCCCAAATCATTCACAACAAC	57.4	148
*MVD*	NM_002461.2	GGTGGCACCTGTTCTTCTCTCT	CTGATGAGCAGCTGTCTGGAGT	56.5	82
*MVK*	NM_000431.3	CTGGACACAAGCTTTCTGGA	AAGCCTGCAACCTCCTTTAG	57.7	83
*PEX3*	NM_003630.2	TTCTTTTGCGGGTCCAGTTA	ACATCTGGGGGAGCAAGAAT	57.4	100
*PEX5*	NM_001131023.1	CTGAGGCAGTGAGTGTTCTT	TCAGCCACCAACTCATCTTC	57.5	100
*PEX6*	NM_000287.3	AACAGTTGGGGAAGCTCCAG	ATGGAACAGGGCTCAGGGTA	59.9	101
*PEX7*	NM_000288.3	CTCAGGAGGTGTATAGTGTTGATT	CAGTTGGATCCCACAATTTGAC	57.8	99
*PEX10*	NM_153818.1	TGGAGTGGAGGAAGGAGGTT	GATGGGTCCACCTGGATGAT	59.8	118
*PEX11alpha*	NM_003847.2	GGTAATGAAGCTCAAGAAACTGGAG	TGCTCTGCTCAGTTGCCTGT	59.9	101
*PEX11beta*	NM_003846.2	CCAGTCCTGAGTTACAGAAACAGATT	TGACTCAAGGGCATCTGCTG	60.7	101
*PEX11gamma*	NM_080662.3	ACCGCCTGATCCGAGTG	CATCAAAGAGTCGCAAGATGGT	58.7	150
*PEX12*	NM_000286.2	AAGCTCTGGAGCACAAACCA	ACACCCCCAACAGCTTTCTT	59.8	103
*PEX13*	NM_002618.3	CCATGTAGTTGCCAGAGCAG	CATCAAGGCTAGCCAGAAGC	58.3	140
*PEX14*	NM_004565.2	CTGCCTTTGGCTTTGATCTC	CGTGGTGTCACGGTAGTCAA	57.1	137
*PEX16*	NM_004813.2	CGAGCTGTCAGAGCTGGTGTACT	ACAGCGACACAGGCAACTTTT	64.1	101
*PEX19*	NM_002857.3	CTCTCAGAGGCTGCAGGGAG	GTGGCATTTTTGGCTAATCCA	61.7	101
*PMVK*	NM_006556.3	GCCTTTCGGAAGGACATGAT	GTCACTCACCAGCCAGATG	58	114
*PPARalpha*	NM_005036.4	CTGGCCAAGAGAATCTACGAG	ACTGGTTCCATGTTGCCAAG	57.9	
*PPARbeta*	NM_177435.2	AACATGCAAGGCACTGACTG	CTGCCAAAGTGCTGGGATT	59	129
*PPARgamma*	NM_138712.3	ATCTTTCAGGGCTGCCAGT	TCGTGGACTCCATATTTGAGG	58.9	131
*PRDX6*	NM_004905.2	TTAGTGCCATGTGCCTTTCA	TAGCAACCCACTGCAAGAAG	57.7	144
*PSP/SPLUNC2*	NM_001319164.1	GAAGTCTGAGGTGGTGTCAAG	TGCCAAGATTGTCAAGAAGAGA	58.2	107
*RPL13*	NM_000977.	CGGAATGGCATGGTCTTGA	CCTTACGTCTGCGGATCTTAC	57.8	100
*SOD1*	NM_000454.4	AGGATGAAGAGAGGCATGTTG	ATGGTCTCCTGAGAGTGAGAT	57.7	107
*SOD2*	NM_000636.2	GTTGGCCAAGGGAGATGTTA	CGTTAGGGCTGAGGTTTGT	57.5	110
*SQS*	NM_001287742.1	GAAGTCAGTGAGACCAAGAACC	CGCTCTCTGTAGAGCCTTAGA	58.6	76
*TBP*	NM_003194.4	TGACCCAGCATCACTGTTTC	GCTGGAACTCGTCTCACTATTC	58.1	118
*Thiolase*	NM_001607.3	GATGCCTTCTTACCCCAACA	CCCAACCACTGCATAAGACC	57.5	
*TRX1*	NM_001244938.1	GGACGCTGCAGGTGATAAA	CACTCTGAAGCAACATCATGAAAG	57.9	102
*TRX2*	NM_012473.3	GTTAGAGAAGATGGTGGCCAAG	GCTGACACCTCATACTCAATGG	58.7	99

## Data Availability

The data used to support the findings of this study are available from the corresponding author upon request.
